# Syncope due to non-sustained episodes of *Torsade de Pointes* associated to androgen-deprivation therapy use: a case presentation

**DOI:** 10.1186/s12872-021-01945-3

**Published:** 2021-03-12

**Authors:** Ximena Morales, Diego Garnica, Daniel Isaza, Nicolas Isaza, Felipe Durán-Torres

**Affiliations:** 1 School of Medicine and Health Sciences, Internal Medicine Program, Fundación Cardioinfantil, Universidad del Rosario, Carrera 24 #63C-69, Bogotá, Colombia; 2 Fundación Cardioinfantil, Universidad del Bosque, Bogotá, Colombia; 3 Division of Cardiology, Fundación Cardioinfantil, Bogotá, Colombia; 4 Department of Internal Medicine, Beth Israel Deaconess Medical Center, Boston, MA USA; 5 School of Medicine and Health Sciences, Public Health Research Group, Universidad del Rosario, Bogotá, Colombia

**Keywords:** Prostatic neoplasms, Abiraterone acetate, Leuprolide, Androgen-deprivation, Ventricular tachycardia, Hypokalemia, Case presentation

## Abstract

**Background:**

Abiraterone is a medication frequently used for metastatic castrate-resistant prostate cancer. We report a case of non-sustained episodes of TdP associated with severe hypokalemia due to androgen-deprivation therapy. Few case presentations describe this association; the novelty lies in the potentially lethal cardiovascular events among cancer patients receiving hormonal therapy.

**Case presentation:**

A 70-year-old male presented with recurrent syncope without prodrome. ECG revealed frequent ventricular ectopy, non-sustained episodes of TdP, and severe hypomagnesemia and hypokalemia. During potassium and magnesium infusion for repletion, the patient underwent temporary transvenous atrial pacing. As part of the work-up, coronary angiography revealed a mild coronary artery disease, and transthoracic echocardiogram showed a moderately depressed ejection fraction. After electrolyte disturbances were corrected, the QT interval normalized, and transvenous pacing was no longer necessary. Abiraterone was discontinued during the admission, and the patient returned to baseline.

**Conclusions:**

Cancer treatment is complex and requires a multidisciplinary approach. We presented a case of non-sustained TdP associated with androgen-deprivation therapy in an elderly patient with mild coronary artery disease and moderately reduced ejection fraction. Close follow-up and increased awareness are required in patients with hormonal treatment, especially in the setting of other cardiovascular risk factors.

## Background

The use of androgen deprivation therapy represents a milestone in treating both castrate sensitive and castrate-resistant metastatic prostate cancer [1–3]. Dual pharmacologic therapy with medications such as leuprolide and abiraterone acetate is directed towards minimizing testosterone levels and blocking the androgen receptors [4]. Abiraterone acetate plus prednisone has demonstrated a reduction in all-cause mortality compared to placebo in randomized controlled clinical trials [5].

As is the case with other cancer treatment types, such as chemotherapy, immunotherapy, and radiotherapy, androgen deprivation treatment is associated with cardiovascular complications [6, 7]. Abiraterone is usually associated with adverse metabolic effects as hyperglycemia, hyperlipidemia, and hypertension. The latter is related to a strong mineralocorticoid effect, which also produces hypokalemia [8]. To prevent this undesired effect prednisone is prescribed along with abiraterone acetate to reduce the incidence of hyperaldosteronism. Despite the use of prednisone, recent studies have reported an increased incidence of cardiovascular events in real-world patients receiving abiraterone acetate and leuprolide [9]. Furthermore, adverse events as ventricular tachycardia and acquired long QT syndrome-related to abiraterone are scarcely reported. In 2019, the Food and Drug Administration (FDA) published a potential signal of serious risks about abiraterone and is currently evaluating the need for regulatory action; however, no official statement has been released [10].

We report a case of acquired long QT syndrome complicated with non-sustained Torsades de Pointes ventricular tachycardia associated with androgen-deprivation therapy.

### Case presentation

A 70-year-old man with a past medical history of hyperlipidemia, hypertension, and castrate-resistant metastatic prostate cancer, presented to the emergency room with recurrent syncope episodes without prodrome, with some episodes occurring in the decubitus position. Medications included abiraterone acetate 1000 mg BID, monthly 7.5 mg injection of leuprolide, losartan 50 mg BID, and metoprolol succinate 12.5 mg QD. Of note, the patient was not receiving prednisone and did not report any chest pain, palpitations, dyspnea, or any other associated symptoms.

**Fig. 1:**
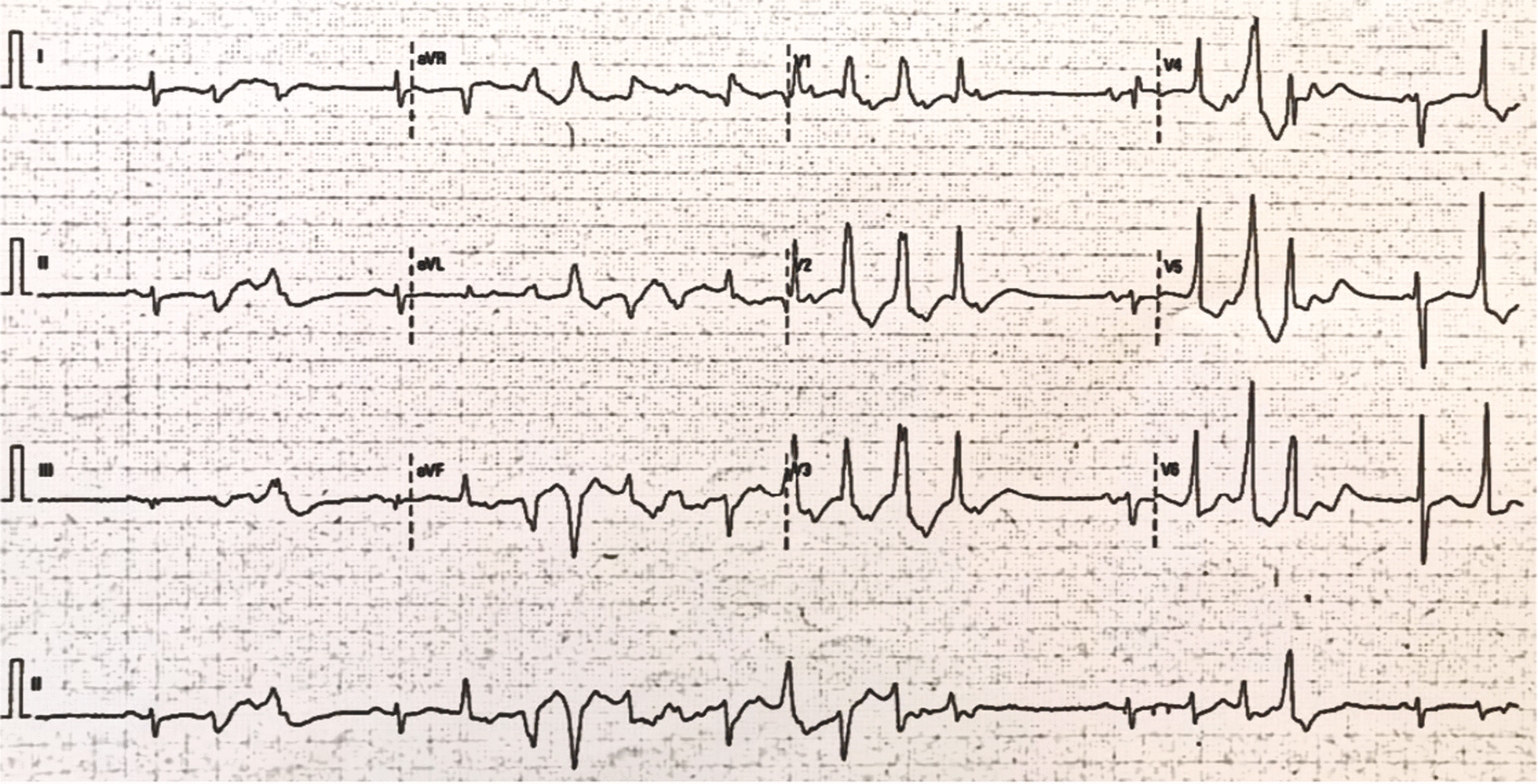
Non sustained TdP

**Fig. 2:**
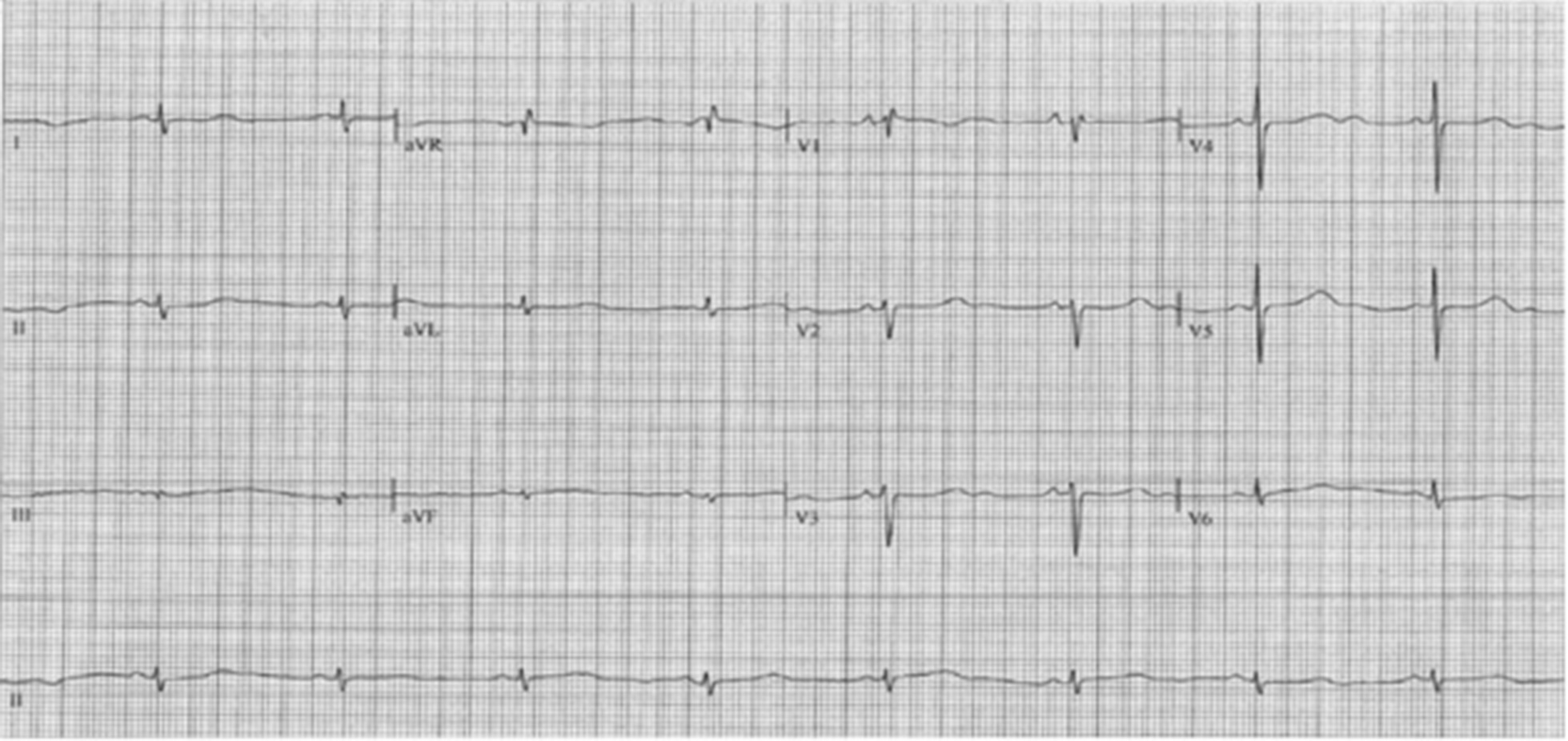
Sinus rhythm, with long QTc: 526 msg (calculated with Bazett’s formula)

Physical exam was notable for a blood pressure of 148/92 mmHg, cardiac auscultation with irregular beats. Presentation ECG showed sinus rhythm with a QTc interval of 580 ms (calculated with Bazett’s formula), frequent premature ventricular beats, and short runs of non-sustained TdP. (Figs. 1, 2). Laboratory results showed severe hypokalemia 2.4 mEq/L (reference range 3.5–5.0 mEq/L), severe hypomagnesemia 0.8 mg/dl (Reference range 1.6–2.6 mg/dl), and preserved renal function (creatinine 0.6 mg/dL and blood urea nitrogen was 10 mg/dL) for an estimated GFR of 102/mL/min/1.73 m^2^ (calculated by CKD-EPI formula).

**Fig. 3:**
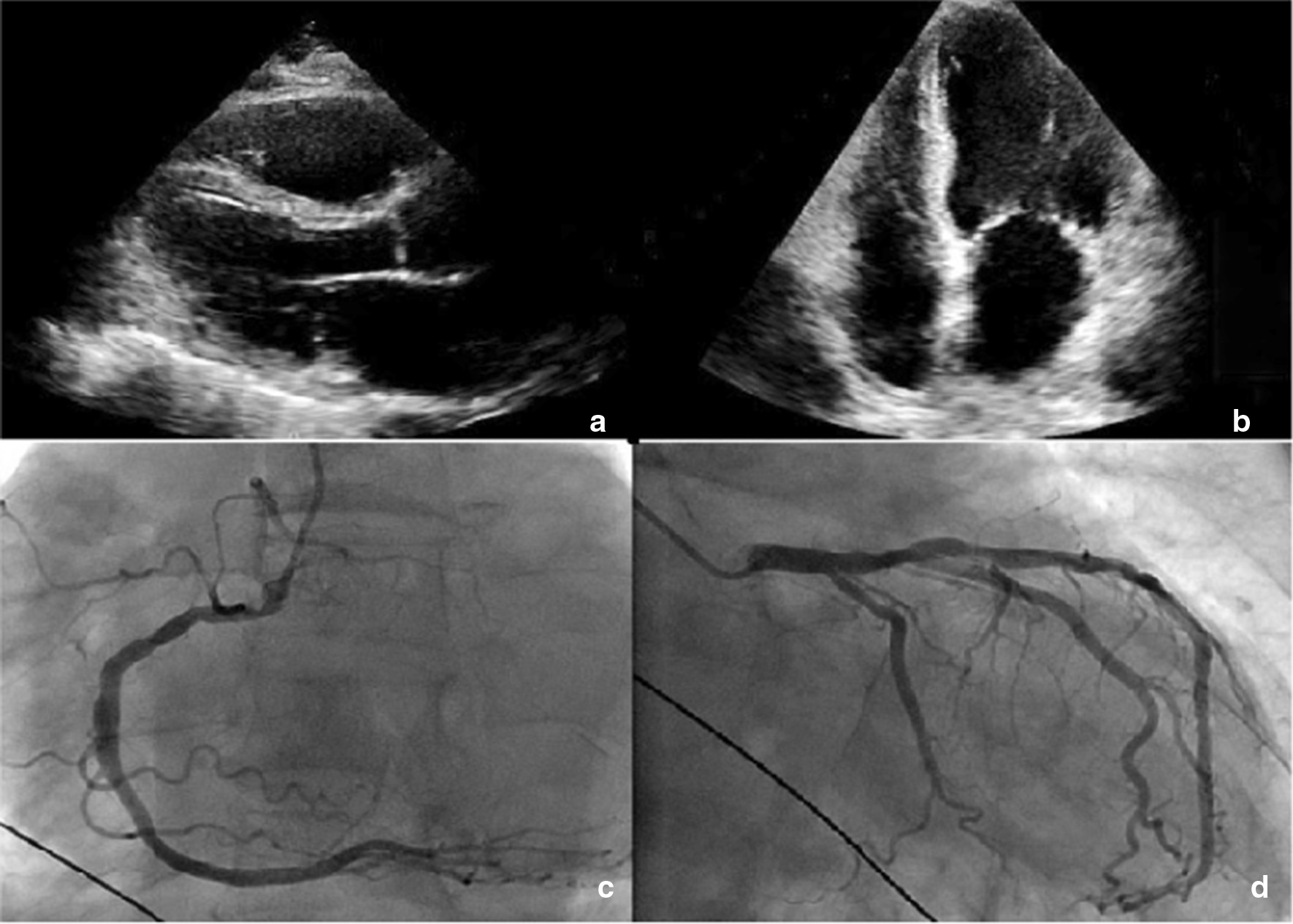
**a**, **b** Transthoracic echocardiogram parasternal long axis view and apical 4 chamber view. **c**, **d** coronary artery angiography showed non-obstructive mild lesions in the left anterior descending artery and the right coronary artery

The patient was admitted to the coronary care unit for continuous telemetry, electrolyte repletion, and temporary transvenous atrial pacing to suppress ectopy and prevent polymorphic ventricular tachycardia. Transthoracic echocardiogram showed a moderate depressed left ventricular ejection fraction of 38% with global hypokinesis (Fig. 3). Coronary angiography revealed mild lesions in the left anterior descending and the right coronary artery. The ventricular dysfunction was thought to be secondary to arrhythmia induced cardiomyopathy rather than the primary cause of the arrhythmia. After a thorough review of causes of hypokalemia (including medication review, metabolic alkalosis, and gastrointestinal losses), abiraterone acetate was suspected to be the cause and was suspended; this was particularly high on the differential given that the patient was not taking prednisone and the use of abiraterone acetate alone can result in a clinical picture similar to hyperaldosteronism. As potassium and magnesium levels normalized, the QTc interval shortened, the ventricular ectopy and runs of non-sustained episodes of TdP were entirely resolved. The patient was eventually transferred to the general ward and was later discharged after three days of being asymptomatic with no telemetry abnormalities.

The patient stopped taking abiraterone acetate and no further electrolyte disturbances were presented, suggesting no underlying genetic abnormalities were the main cause of the arrythmia, such as is the case of Gitelman syndrome, which could have explained the severe hypokalemia and hypomagnesemia (least likely because of the absence of metabolic alkalosis and hypertension medical history).

## Discussion and conclusions

Abiraterone inhibits androgen synthesis and is approved by the FDA for castrate-resistant prostate cancer [1, 2]. Cancer therapies are known to be associated with cardiovascular and other side effects [6]. In particular, abiraterone causes hypertension and hypokalemia in 32% and 20% of cases, respectively [5, 8]. However, cardiovascular severe adverse events like long QT and ventricular tachycardia are rarely reported [11, 12]. This cardiovascular event is probably due to the association between androgen deprivation and lower testosterone levels that result in iatrogenic Hypogonadism [13, 14]. A recent study found that testosterone plays an essential role in cardiac repolarization by altering repolarization currents (increasing the repolarizing currents IKr and IKs, and decreasing the depolarizing current ICaL) [15]. This iatrogenic Hypogonadism induced by medications such as abiraterone and leuprolide has been associated with long QTc and TdP [15], even to the point of suggesting androgen deprivation therapy as the second cause of drug-induced long QTc, particularly with abiraterone [16]. It is essential to highlight that leuprolide itself produces and reinforces this hypogonadism state, making an additive effect of these cardiovascular events when it is combined with abiraterone [15].

Androgen deprivation has been associated with cardiotoxicity, including QT prolongation [9, 17, 18]. In 2019, the FDA published a potential signal of serious risks about abiraterone and is currently evaluating the need for regulatory action; nonetheless, no official statement has been released yet [10]. In this setting, we present a case of acquired long QT syndrome complicated with non-sustained Torsades de Pointes ventricular tachycardia associated with severe hypokalemia and hypomagnesemia attributed to the use of abiraterone acetate without prednisone and was enhanced by the concomitant leuprolide use [15]. Furthermore, this acute episode resulted in arrhythmia induced cardiomyopathy that reverted after correction of electrolyte abnormalities and discontinuation of the offending agent. In this particular case, the patient presented with other conditions that are associated with abnormal repolarization and TdP, such as coronary heart disease, hypertension, leuprolide use, male gender and advanced age, suggesting that multiple QTc prolonging phenomena are needed to “hit”, before TdP is ensued [19, 20]. We believe that our case represents a heightened risk of acquired long QTc derived from abiraterone acetate and leuprolide use [15].

Despite having the highest risks due to these medications, the data about the use of abiraterone acetate in men older than 65-years-old is insufficient. Data about adverse events in these specific groups is even more limited because pivotal trials excluded patients with pre-existing cardiovascular disorders [5]. Limited evidence includes a cohort study performed between 1991 and 2013 to evaluate if patients with pre-existing cardiovascular disease, using abiraterone or enzalutamide had increased risk of hospitalization and all-cause mortality. In this study, there was an association between hypertension and the use of abiraterone acetate and higher hospitalization rates [18].

More recently, in 2012, a single-arm open clinical trial of 33 patients taking abiraterone evaluated ECG changes during the first two days of use, finding no significant association with QT prolongation [21]; nevertheless, this is a limited study due to trial design, small sample size, and poor ECG follow-up, limiting the ability to conclude. In 2016 a retrospective analysis of abiraterone use in patients with pre-existing cardiovascular conditions concluded that there was no worsening of cardiovascular diseases, and cardiovascular events more frequently reported were fluid overload and hypertension, without reports of arrhythmias during the follow-up [22]. However, this study also presents limitations mainly related to its retrospective nature. In contrast to the previous two studies, a meta-analysis suggests an increased risk in the pooled occurrence of ischaemic heart disease, myocardial infarction, supraventricular tachyarrhythmias, ventricular tachyarrhythmias, heart failure associated with abiraterone acetate [23]. Finally, there are anecdotal reports of abiraterone acetate resulting in QT prolongation and life-threatening ventricular arrhythmias, similar to our case [11, 12]. Nonetheless, all the studies mentioned above have significant limitations, and further research is required.

Increased follow-up and awareness are required in patients with androgen deprivation therapy to evaluate the presence of QT prolongation and ventricular arrhythmias arising from electrolyte disorders.

## Data Availability

Data sharing does not apply to this article as no datasets were generated or analyzed during the current study.

## Abbreviations

TdP: *Torsade de Pointes*
ECG: Electrocardiogram
QTc: QT interval corrected for heart rate
FDA: Food and Drug Administration
BID: bis in die (which means, in Latin, twice a day)
QD: quaque die (which means, in Latin, every day/daily
